# Diagnostic Accuracy of the Recognizing Acute Delirium as Part of Your Routine (RADAR) Scale for Delirium Assessment in Hospitalized Older Adults: A Cross-Sectional Study

**DOI:** 10.3390/healthcare12131294

**Published:** 2024-06-28

**Authors:** Diletta Fabrizi, Paola Rebora, Valentina Spedale, Giulia Locatelli, Giuseppe Bellelli, Stefania Di Mauro, Davide Ausili, Michela Luciani

**Affiliations:** 1 Department of Medicine and Surgery, University of Milano-Bicocca, 20900 Monza, Italy; diletta.fabrizi@unimib.it (D.F.); paola.rebora@unimib.it (P.R.); giulia.locatelli@unimib.it (G.L.); giuseppe.bellelli@unimib.it (G.B.); stefania.dimauro@unimib.it (S.D.M.); davide.ausili@unimib.it (D.A.); michela.luciani@unimib.it (M.L.); 2 Bicocca Bioinformatics Biostatistics and Bioimaging (B4) Centre, School of Medicine and Surgery, University of Milano-Bicocca, 20900 Monza, Italy; 3 Biostatistics and Clinical Epidemiology, IRCCS San Gerardo dei Tintori Foundation, 20900 Monza, Italy; 4 Bachelor’s Degree in Nursing Program, IRCCS San Gerardo dei Tintori Foundation, 20900 Monza, Italy; 5 Acute Geriatric Unit, IRCCS San Gerardo dei Tintori Foundation, 20900 Monza, Italy

**Keywords:** delirium, RADAR, hospitalization, older patients, CAM, Nursing Delirium Screening Scale (Nu-DESC), 4 ‘A’s Test (4AT)

## Abstract

Delirium is highly prevalent among hospitalized older adults and is associated with unfavorable outcomes. However, delirium often remains undiagnosed in the hospital context. Having a valid, simple, and fast screening tool could help in limiting the additional workload for healthcare professionals, without leaving delirium undetected. The aim of this study was to estimate the sensitivity and specificity of the Recognizing Acute Delirium As part of your Routine (RADAR) scale in an Italian hospital. An observational cross-sectional study was conducted. A total of 150 patients aged ≥70 years were enrolled. Receiver operating characteristic (ROC) curves using the Confusion Assessment Method (CAM) criterion-defined delirium as the gold standard were plotted to evaluate the performance of the RADAR scale. The cut-off suggested by previous research was used to estimate the sensitivity, specificity, and positive and negative predictive values of the RADAR scale. The involved patients were mostly females (60%; *n* = 90), with a median age of 84 years (I–III quartiles: 80–88). According to the CAM and the RADAR scale, 37 (25%) and 58 (39%) patients were classified as experiencing delirium, respectively. The area under the ROC curve of the RADAR scale was 0.916. Furthermore, the RADAR scale showed robust sensitivity (95%), specificity (80%), and positive (60%) and negative predictive values (98%). The RADAR scale is thus suggested to be a valid tool for screening assessment of delirium in hospitalized older adults.

## 1. Introduction

Delirium is defined as an acute, fluctuating disturbance of attention and cognitive functioning that is almost always triggered by underlying medical causes and is often accompanied by abnormal arousal and perceptual disturbances [1]. It is estimated that 23% of hospitalized adults (≥18 years) have delirium [2], and this prevalence increases ro over 30% in older hospitalized patients (≥70 years) [3]. Indeed, advanced age is an independent risk factor for delirium upon admission or during the length of stay in hospitalized older adults [4]. Postoperative delirium rates among older adults range from 15 to 25% after major elective surgery [3], from 12 to 26% after intracranial surgery [5], and up to 50% after cardiac surgery and hip fracture repair [3]. Among patients of any age admitted to intensive care units (ICUs) and requiring mechanical ventilatory support, the prevalence of delirium reaches 80% [6]. Delirium prevalence is also significant within palliative care environments: prevalence rates are 4–12% in community settings, 9–57% among hospitalized patients, and 6–74% in inpatient palliative care units, with a pooled prevalence of 35% [7]. Additionally, the observed delirium prevalence was estimated to be between 59 and 88% among palliative care inpatients in the final weeks before death [8].

Delirium is associated with many adverse clinical outcomes, including a prolonged intensive care unit and hospital stay [3,9], increased mortality rates [10], cognitive decline [11], impaired cognitive function [12,13], restrictions in motor functionality [14], an ongoing need for care in long-term care institutions [9], and an increased likelihood of discharge to destinations other than home [15]. From an economic perspective, delirium is strongly associated with additional healthcare costs [16] and greater burdens on healthcare professionals [17,18].

Although delirium is a common condition, 55–80% of cases are unrecognized and undocumented by the treating clinical team [19]. One potential explanation for this occurrence may be that delirium receives various clinical labels (e.g., acute confusion, acute organic brain syndrome, brain failure, psychosis, toxic encephalopathy, etc.) which hinder appropriate communication among healthcare professionals and do not help to promote knowledge about delirium [20]. Another reason may be that medical culture does not regard delirium as a potentially lethal condition and, consequently, delirium is not actively searched for in routine clinical practice [20]. However, several tools exist to recognize and assess delirium [21].

The Confusion Assessment Method (CAM) [22] is one of the most widespread tools [23], showing high sensitivity and excellent specificity [21,23,24,25]. The CAM takes five to ten minutes to be administered. However, special training is required to use it properly for optimal performance, which may represent a limitation to its systematic use [26]. Furthermore, the combination of observation-based and interactive items makes it less feasible for use by nurses in daily practice due to time constraints [21]. Over time, several easier and faster-to-use screening tools have been developed to facilitate the systematic assessment of delirium in clinical settings, including the Nursing Delirium Screening Scale (Nu-DESC) [27], the 4 ‘A’s Test (4AT) [28], and the Recognizing Acute Delirium As part of your Routine (RADAR) scale [29].

The Nu-DESC is a delirium screening tool designed to be used by nurses, according to their observations [27]. It takes less than two minutes to be completed [21,27]. The 4AT is a short delirium screening assessment tool designed for routine use that takes around two minutes to be administered and does not require special training [28,30]. Both the Nu-DESC [31] and the 4AT [28] have been validated in Italian geriatric settings and showed good diagnostic accuracy [21,23]. The RADAR scale is a screening tool designed to be completed by nurses during the scheduled distribution of medications in an average of seven seconds [29]. Consequently, assessing the presence of delirium four times a day requires less than one minute overall. The RADAR scale showed good diagnostic accuracy, supporting its use among older patients and residents with or without cognitive impairment [29].

As screening is crucial for enhancing the identification and timely treatment of delirium [32], the presence of an accurate, simple, and fast screening tool that can be used during routine activities could minimize any additional workload for nurses without leaving delirium cases undetected. Although the RADAR scale has been proven to have these characteristics, its applicability and diagnostic accuracy have never been tested in the Italian context.

Thus, the primary aim of this study was to determine the sensitivity and specificity of the RADAR scale in assessing delirium among hospitalized older adults in the Italian context, using CAM criterion-defined delirium as the gold standard. The two secondary aims were (1) to evaluate the sensitivity and specificity of the Nu-DESC and 4AT, using CAM criterion-defined delirium as the gold standard and (2) to compare the sensitivity and specificity of the RADAR scale, Nu-DESC, and 4AT.

## 2. Materials and Methods

An observational cross-sectional study was conducted in a geriatric unit of an acute care hospital in northern Italy.

### 2.1. Sample

Patients aged 70 years or older for whom medication administration was planned were enrolled in the present study through a convenience sampling method. Individuals with a history of psychiatric illness, specifically psychotic disorders, bipolar disorders, and major depressive disorders with psychotic features were excluded. Patients with severe cognitive impairment documented in their medical records were also excluded.

### 2.2. Measurements

The presence of delirium was assessed using the CAM [22] as the gold standard. The CAM is recognized as one of the most effective bedside screening tools for this purpose [21,23,24,25,26]. It was developed according to the Diagnostic and Statistical Manual of Mental Disorders (DSM-III-R) [33] and validated in comparison with expert opinion [22]. To ensure optimum diagnostic accuracy, specific training for the operators who administer the test is required [26]. As anticipated above, the CAM combines observational and interactive items assessing four conditions: (1) an acute onset and fluctuating course of the disorder; (2) inattention or distractibility; (3) disorganized thinking; and (4) an altered level of consciousness. The diagnosis of delirium is obtained in the presence of condition 1 and 2 and either 3 or 4 [22]. In its validation study, the CAM showed a sensitivity of 94–100% and a specificity of 90–95% [22].

Delirium was also screened with the Nu-DESC [27], which consists of five items evaluating disorientation, inappropriate behavior, inappropriate communication, illusions/hallucinations, and psychomotor retardation. Each item is scored on a 0 (no symptoms) to 2 (maximum symptomatology) scale [27], and a total score equal to or higher than 3 is considered as suggesting the presence of delirium [31]. In its validation study, the Nu-DESC showed a sensitivity of 86% and a specificity of 87% in comparison with the CAM evaluation [27].

Another tool used for the screening of delirium was the 4AT [28]. The 4AT consists of four items. Item 1 evaluates the level of alertness [34]. Item 2 coincides with the Abbreviated Mental Test—4 (AMT4) [35], which evaluates the patient’s ability to recall their age, date of birth, the place, and the current year. Item 3 tests attention by listing the months backwards [36]. Item 4 assesses acute changes or fluctuations in mental status [1]. The 4AT is scored on a 0–12 scale. A score equal to or higher than 4 is considered positive for delirium, while scores between 1 and 3 suggest possible cognitive impairment [28]. The 4AT had a sensitivity of 89.7% and specificity 84.1% for delirium in comparison with the diagnosis provided by an expert assessor according to the Diagnostic and Statistical Manual of Mental Disorders (DSM-IV-TR) [28,37].

The RADAR scale was also used for the screening of delirium. The RADAR scale consists of three items asking the following: “When you gave the patient his/her medication: (1) Was the patient drowsy? (2) Did the patient have trouble following your instructions? (3) Were the patient’s movements slowed down?”. Each item requires a dichotomous Yes/No response. A positive screening is determined if at least one item is marked as “Yes” [29]. The RADAR scale had a sensitivity of 73% and a specificity of 67% in comparison with DSM-IV-TR criterion-defined delirium [29].

The Charlson Comorbidity Index (CCI) [38] was used to assess comorbidities. The scoring algorithm of the CCI attributes different weights (1, 2, 3, or 6) to the presence of each one of 19 different medical comorbid conditions, according to the specific adjusted risk of 1-year mortality [19]. Thus, the total score is obtained by summing the weights, with higher scores denoting both increasingly severe comorbid conditions and a higher mortality risk [38,39].

Lastly, the New Mobility Score (NMS) [40] was used to evaluate the functional status, in reference to one-month prior hospitalization. The NMS assesses, through three items, individuals’ ability to walk both indoors and outdoors, as well as their capacity to independently shop for themselves. [40]. Each item is scored on a 0–3 scale, with higher NMS total scores meaning higher functional status and scores lower than 6 indicating functional impairment [40,41].

### 2.3. Data Collection

The data were collected between November 2017 and February 2020 by two research assistants (RAs). The first RA, referred to as RA-Delirium, was responsible for utilizing the CAM, 4AT, and Nu-DESC to assess the presence of delirium. Moreover, the RA-Delirium gathered socio-demographic and clinical data. The second RA, named RA-RADAR, conducted the RADAR assessment within 30 min of RA-Delirium’s evaluation, while being unaware of the delirium status determined by the CAM, 4AT, and Nu-DESC assessments. In addition, RA-RADAR administered the NMS and the CCI. Both research assistants received training in using the CAM.

The following socio-demographic and clinical characteristics were collected from medical records: gender, age, school education, family support, diagnosis of delirium, and number of medications.

### 2.4. Statistical Analyses

Socio-demographic and clinical data were described with frequencies and percentages when variables were categorical and with median and quartiles when continuous. Socio-demographic and clinical data were compared using the exact Fisher test or the Mann–Whitney U test, as appropriate.

Receiver operating characteristic (ROC) curves using CAM criterion-defined delirium as the gold standard were plotted to evaluate the performance of the RADAR, Nu-DESC and 4AT. The area under the ROC curve (AUC) was also calculated. The cut-off points recommended by previous research [29,31,41] were applied for each scale to determine the sensitivity (SE), specificity (SP), positive predictive value (PPV), and negative predictive value (NPV) with relevant 95% exact binomial confidence intervals (CI). The binomial one-sample test was used to assess whether the sensitivity of the RADAR scale was higher than 70% and the specificity was higher than 60% (one-tailed test, type 1 error of 0.05). Analyses were performed in R (R Core Team, 2023, R Foundation for Statistical Computing, Vienna, Austria).

### 2.5. Ethical Approval and Informed Consent

The study procedures were carried out in compliance with the ethical guidelines set by the responsible committee for human experimentation (both at the institutional and national levels) and adhered to the principles outlined in the 1964 Helsinki Declaration, along with its subsequent amendments. The study was approved by the Institutional Review Board “Brianza Ethics Committee”. Written informed consent was obtained from all participants involved in this study.

## 3. Results

A total of 150 patients were enrolled in this study. Table 1 presents the socio-demographic and clinical characteristics of the overall sample and the subsets of patients identified as delirium-negative and delirium-positive based on the CAM. The overall sample was mostly composed of females (60.0%; *n* = 90), with a median age of 84 years (I–III quartiles, Q1–Q3 = 80, 88). The vast majority had a low level of education (i.e., none, elementary or middle school: 82%; *n* = 123) and had family support (73%; *n* = 110). The median number of medications taken by patients was 10 (Q1–Q3 = 8, 13), the median CCI score was 3.5 (Q1–Q3 = 3.0, 5.8), and the median NMS was 3 (Q1–Q3 = 1.2, 6.0).

Based on the CAM, 37 (25%) patients were diagnosed with delirium. CAM delirium-positive patients showed lower education levels (*p* = 0.004) and lower functional statuses (*p* = 0.007) than CAM delirium-negative patients.

**Figure 1:**
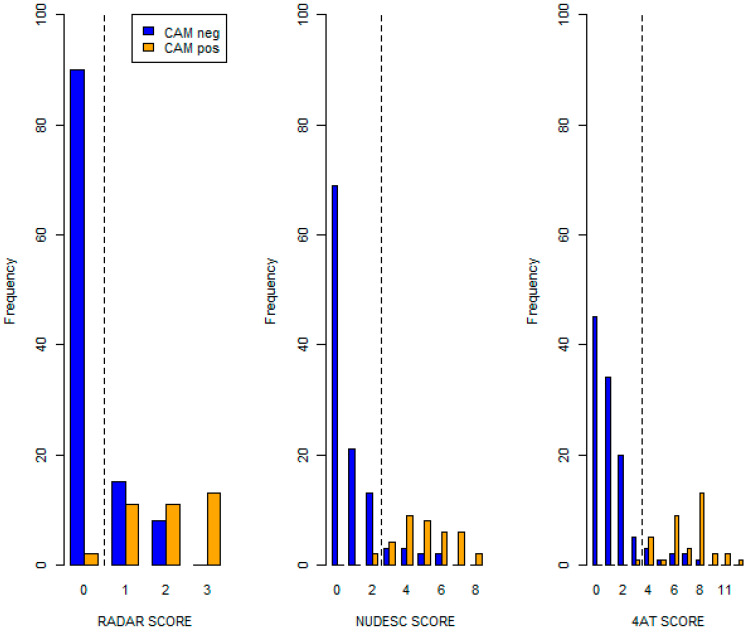
Distribution of the delirium assessment tool scores versus CAM criterion-defined delirium as the gold standard (*n* = 150) (dashed line reports the cut-off value for each score).

A total of 58 (39%) patients were diagnosed with delirium based on the RADAR scale, 45 (30%) were diagnosed using the Nu-DESC, and 45 (30%) were diagnosed using the 4AT. Figure 1 shows the distributions of the RADAR scale and the other delirium scales according to the gold standard (CAM).

Patients classified as delirium-negative by CAM had lower values in the three scales with respect to the patients classified as delirium-positive (Table S1 in Supplementary Materials: Scores in delirium assessment tools by the presence of CAM criterion-defined delirium).

**Figure 2:**
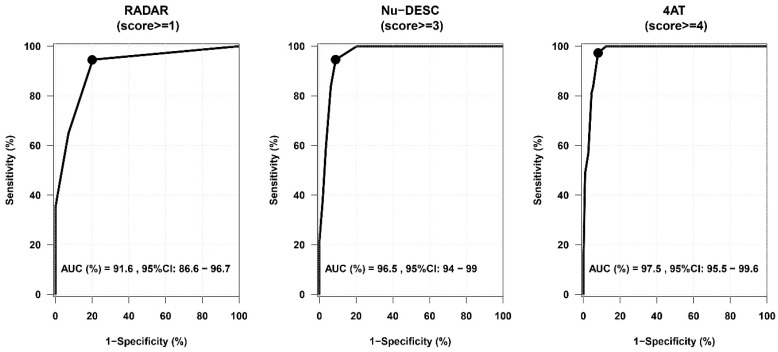
ROC curves of delirium assessment tools versus CAM criterion-defined delirium as the gold standard (CI = confidence interval).

The ROC curve of the RADAR scale (using CAM criterion-defined delirium as the gold standard) with an AUC of 0.916 (95% CI = 0.866–0.967) is presented in Figure 2.

By using the suggested cut-off score of 1, sensitivity was higher than 70% (*p* = 0.002034) and equal to 95% (95% CI = 82%, 99%). The RADAR scale’s specificity was 80% (95% CI = 71, 87%), its PPV was 60%, and its NPV was 98% (Table 2). The ROC curves of Nu-DESC and 4AT are also presented in Figure 2, and both showed an AUC higher than 94%. The 4AT (≥4) showed the highest sensitivity (97%, 95% CI = 86, 100%), specificity (92%, 95% CI = 85, 96%), PPV (80%), and NPV (99%) compared to the 4AT and RADAR scale, while the 4AT showed the largest AUC: 0.975 (95% CI = 0.955, 0.996). All diagnostic accuracy parameters of the RADAR scale, Nu-DESC, and 4AT are presented in Table 2.

## 4. Discussion

The primary aim of this study was to estimate the diagnostic accuracy of the RADAR scale for delirium in hospitalized older adults in the Italian context. To the best of our knowledge, this is the first study estimating the sensitivity and specificity of the RADAR scale in the Italian context. Moreover, this is the first Italian study assessing the diagnostic accuracy of the RADAR scale, Nu-DESC, and 4AT in the same patient sample. We found that the RADAR scale had a sensitivity higher than 70% and a specificity higher than 60%. In its first validation study [29], the RADAR scale showed a sensitivity of 73% and a specificity of 67% using CAM criterion-defined delirium as the gold standard. Compared to that study, our data indicated that it has both higher sensitivity (95%) and specificity (80%). This result could be partly due to 21% of patients in the first validation study having dementia [29], while patients with severe cognitive impairment were excluded from our sample.

The sensitivity and specificity values shown by the RADAR scale in the present study are in accordance with the general expectations of a screening tool [42]: to achieve high sensitivity (i.e., identifying the largest number of patients with delirium) while guaranteeing adequate clinical specificity (i.e., identifying the smallest number of false positives). In addition, the RADAR scale showed a PPV of 60%, suggesting its use as the first screening tool for the presence of delirium and then performing more accurate diagnostic tests (e.g., CAM, Nu-DESC or 4AT) only in positive cases [42]. Indeed, the systematic utilization of the RADAR scale does not significatively impact the workload of nursing staff, as it requires a few seconds and can be completed during already scheduled routine activities. Furthermore, while the routine use of the RADAR scale does not leave delirium cases undetected, it can limit the administration of more comprehensive diagnostic tools to positive cases to exclude potential false positives. This is more time-efficient, acceptable, and feasible than systematically screening all patients with more complex tools.

According to the secondary aims of the present study, the sensitivity and specificity of the Nu-DESC and the 4AT were tested, confirming the diagnostic accuracy of both of these scales. In the previous Italian Nu-DESC validation study [31], the sensitivity was 100%, the specificity was 76%, and the plotted ROC curve displayed an AUC of 0.94. In the present study, the Nu-DESC showed lower sensitivity (95%) but higher specificity (91%) and a larger AUC (0.965). In this study, the 4AT reported higher sensitivity (97%), specificity (92%), and AUC values (0.975) than those estimated in the previous Italian validation study [28]. Indeed, in the previous Italian validation study [28], the 4AT’s sensitivity was 89.7%, its specificity was 84.1%, and its AUC was 0.93.

Comparing the sensitivity and specificity of the RADAR, Nu-DESC, and 4AT, the 4AT performed better than the other scales in recognizing people with delirium. The RADAR scale showed inferior performance compared to the Nu-DESC and the 4AT but, as previously discussed, it can be very useful as the first screening tool due to its comparable sensitivity and its PPV.

Our findings could be useful both nationally and internationally. In the Italian context, the validation of the RADAR scale provides healthcare professionals with a reliable and efficient tool for initial delirium screening in older hospitalized patients. The availability of the RADAR scale could lead to its widespread adoption across Italian hospitals, enhancing the early detection and management of delirium, ultimately improving patient outcomes [9,12,14] and reducing healthcare costs associated with undiagnosed or late-diagnosed delirium [16]. Internationally, our study contributes to the growing body of evidence supporting the need for the early detection of delirium and the utilization of the RADAR scale in various healthcare settings [43,44,45]. The high sensitivity and specificity observed in our study suggest that the RADAR scale may have high diagnostic accuracy in different populations and settings, encouraging further research and validation studies worldwide. Future applications of these results could include integrating the RADAR scale into international clinical guidelines for delirium assessment and exploring its utility in diverse cultural and healthcare contexts.

### Limitations and Strengths

The limitations of this study include patient recruitment from a single ward within an acute care hospital, which could impact the generalizability of our results. Another relevant limitation coincides with the single delirium assessment for each enrolled patient. Indeed, since delirium is known to be a fluctuating condition, it would have been appropriate to measure it multiple times within a 24-h period. Despite the relatively small sample size, the study was able to demonstrate that the RADAR had a sensitivity higher than 70% and a specificity higher than 60%. The main strength of this study lies in the rigor of the data collection process, as the RA-RADAR was blinded to the delirium status as determined by the CAM, 4AT, and Nu-DESC.

## 5. Conclusions

The present study validates the RADAR scale as a simple, fast, and accurate tool for the screening assessment of delirium, suggesting its utility in clinical practice in the Italian context. Its simplicity stems from the lack of nurse training or patient collaboration required; moreover, it imposes no additional workload on nurses as it aligns with routine therapy administration. In case of RADAR scale positivity, further evaluation of delirium with other scales such as the CAM, NU-DESC, or 4AT is recommended. Future multicentric studies with multiple RADAR assessments are needed to corroborate the findings of the present study.

## Figures and Tables

**Table 1:** Socio-demographic and clinical characteristics of the overall sample and the delirium-negative and delirium-positive patients as measured by the CAM (*n* = 150).

Variable	Overall (*n* = 150)	Delirium-Negative Patients (*n* = 113)	Delirium-Positive Patients (*n* = 37)	*p*
**AGE** (years) Median [1st–3rd quartile]	84 [80, 88]	83 [79, 88]	84 [83, 89]	0.077
**BIOLOGICAL SEX-MALES** (%)	60 (40)	48 (42)	12 (32)	0.336
**SCHOOL EDUCATION** (%)				0.004
None	14 (9)	5 (4)	9 (24)	
Elementary	76 (51)	56 (50)	20 (54)	
Middle School	33 (22)	28 (25)	5 (14)	
High School	21 (14)	19 (17)	2 (5)	
Degree	6 (4)	5 (4)	1 (3)	
**PRESENCE OF FAMILY SUPPORT** (%)	110 (73)	81 (72)	29 (78)	0.523
**NUMBER OF MEDICATIONS** Median [1st–3rd quartile]	10 [8, 13]	10 [7, 13]	10 [8, 13]	0.572
**NEW MOBILITY SCORE (NMS)** Median [1st–3rd quartile]	3.0 [1.2, 6.0]	4.0 [2.0, 6.0]	2.0 [1.0, 4.0]	0.007
**CHARLSON COMORBIDITY INDEX (CCI)** Median [1st–3rd quartile]	3.5 [3.0, 5.8]	3.0 [2.0, 5.0]	4.0 [3.0, 6.0]	0.403

**Table 2:** Diagnostic accuracy parameters of delirium assessment tools versus CAM criterion-defined delirium as the gold standard.

	RADAR (Score ≥ 1)	Nu-DESC (Score ≥ 3)	4AT (Score ≥ 4)
True positive	35	35	36
False positive	23	10	9
True negative	90	103	104
False negative	2	2	1
	Estimate (95% CI)		
Sensitivity	95% (82, 99)	95% (82, 99)	97% (86, 100)
Specificity	80% (71, 87)	91% (84, 96)	92% (85, 96)
Positive predictive value	60% (47, 73)	78% (63, 89)	80% (65, 90)
Negative predictive value	98% (92, 100)	98% (93, 100)	99% (95, 100)

## Data Availability

The data presented in this study are available upon request from the corresponding author.

## Supplementary Materials

The following supporting information can be downloaded at: https://www.mdpi.com/article/10.3390/healthcare12131294/s1, Table S1: Scores in delirium assessment tools by the presence of CAM criterion-defined delirium.
