# Supplementary material for: Diagnostic Accuracy of the Recognizing Acute Delirium as Part of Your Routine (RADAR) Scale for Delirium Assessment in Hospitalized Older Adults: A Cross-Sectional Study
Source: Healthcare (Basel). 2024 Jun 28;12(13):1294. doi: 10.3390/healthcare12131294 (PMC11241281; doi:10.3390/healthcare12131294)
Supplement: Supplementary file 1 [file healthcare-12-01294-s001.zip › healthcare-3012481-supplementary.pdf]

SUPPLEMENTARY MATERIAL

Table S1. Scores in delirium assessment tools by the presence of CAM criterion-defined delir ium

	Delirium by CAM		p
	**No** (n= 113)	**Yes** (n= 37)	
	Median [1st,3rd quartile]		
RADAR	0 [0, 0]	2 [1, 3]	< 0.001
4AT	1 [0, 2]	7 [6, 8]	< 0.001
Nu-DESC	0 [0, 1]	5 [4, 6]	< 0.001

Note. Differences were tested using the Mann-Whitney U test.
